# The effect of a supplementary (‘gist-based’) information leaflet on colorectal cancer knowledge and screening intention: a randomized controlled trial

**DOI:** 10.1007/s10865-014-9596-z

**Published:** 2014-09-25

**Authors:** Samuel G. Smith, Rosalind Raine, Austin Obichere, Michael S. Wolf, Jane Wardle, Christian von Wagner

**Affiliations:** 1 Department of Epidemiology and Public Health, University College London, 1-19 Torrington Place, London, WC1E 6BT UK; 2 Division of General Internal Medicine, Feinberg School of Medicine, Northwestern University, 750 N Lake Shore Drive, Chicago, IL USA; 3 Department of Applied Health Research, University College London, 1-19 Torrington Place, London, WC1E 6BT UK; 4 University College London (NHS) Hospital (UCLH) NHS Trust, 1-19 Torrington Place, London, WC1E 6BT UK

**Keywords:** Fuzzy-Trace Theory, Gist, Colorectal cancer, Screening, Numeracy, Health communication

## Abstract

Guided by Fuzzy Trace Theory, this study examined the impact of a ‘Gist-based’ leaflet on colorectal cancer screening knowledge and intentions; and tested the interaction with participants’ numerical ability. Adults aged 45–59 years from four UK general practices were randomly assigned to receive standard information (‘The Facts’, n = 2,216) versus standard information plus ‘The Gist’ leaflet (Gist + Facts, n = 2,236). Questionnaires were returned by 964/4,452 individuals (22 %). 82 % of respondents reported having read the information, but those with poor numeracy were less likely (74 vs. 88 %, *p* < .001). The ‘Gist + Facts’ group were more likely to reach the criterion for adequate knowledge (95 vs. 91 %; *p* < .01), but this was not moderated by numeracy. Most respondents (98 %) intended to participate in screening, with no group differences and no interaction with numeracy. The improved levels of knowledge and self-reported reading suggest ‘The Gist’ leaflet may increase engagement with colorectal cancer screening, but ceiling effects reduced the likelihood that screening intentions would be affected.

**Electronic supplementary material:**

The online version of this article (doi:10.1007/s10865-014-9596-z) contains supplementary material, which is available to authorized users.

## Introduction

Colorectal cancer is the third most common cancer and fourth most common cause of cancer death worldwide (International Agency for Research on Cancer 2014). It was projected that there would be over 142,000 new cases of colorectal cancer and more than 50,000 deaths in the United States in 2013 (U.S. Cancer Statistics Working Group 2013). Colorectal cancer is also a leading cause of mortality in the United Kingdom (UK), with over 15,000 deaths recorded in 2011 (General Register Office for Scotland 2012; Northern Ireland Statistics Research Agency 2012; Office for National Statistics 2013).

Colorectal cancer screening recommendations vary between countries, but there is evidence for a reduction in cancer-specific mortality with colonoscopy, flexible sigmoidoscopy and the Fecal Occult Blood (FOB) test (Atkin et al., 2010; Hewitson et al., 2007; Whitlock et al., 2008). In comparison with breast and cervical screening, participation rates for colorectal cancer screening are consistently low. Up-to-date colorectal cancer screening prevalence (using any screening modality) for 50–75 year olds was estimated to be 63 % in 2008 (Centers for Disease Control and Prevention 2010b), which is lower than for pap smears in 2010 (18–30 years, up-to-date; 67 %) and considerably lower than mammography (50–74 years, up-to-date; 81 %) (Centers for Disease Control and Prevention 2010a, 2013). In the English organised cancer screening programs there is a consistent 20–25 % gap between the uptake of FOB testing and either breast or cervical screening (Health and Social Care Information Centre 2013; The NHS Information Centre, Screening and Immunisations team 2013).

Participation in all types of colorectal cancer screening is affected by health service, social and individual factors (Power et al., 2009), but there is particular concern about socioeconomic inequalities (Halloran et al., 2012). The likelihood of meeting US colorectal cancer screening recommendations (including colonoscopy, flexible sigmoidoscopy, and FOBt) is consistently lower among disadvantaged groups (Cairns & Viswanath, 2006; Centers for Disease Control and Prevention 2010b; Klabunde et al., 2011; Shapiro et al., 2012). In organised programs where FOBt screening is the primary test, similar patterns occur (Moss et al., 2011; Steele et al., 2010; Szczepura et al., 2008; von Wagner et al., 2009a). Data from the first 2.6 million invitations in the English colorectal cancer screening program showed that uptake of FOBt was 61 % in the least deprived quintile of residential areas but only 35 % in the most deprived quintile (von Wagner et al., 2011).

In England, FOB screening is the primary modality through which the public are tested for colorectal cancer, however a Flexible Sigmoidoscopy program is being implemented. In the current program, test kits are sent biennially through the post to people aged 60–74 years registered with a general practitioner. Tests are sent from the centralised screening program, with no routine contact with a healthcare professional unless a follow-up colonoscopy is recommended. Participation is therefore dependent on the individual’s understanding of the information, motivation to do the test, and capacity to follow the instructions. As a result, there is complete reliance on written materials to convey the aims, practicalities, advantages and disadvantages so that the individual can make an informed decision about participating (Ramirez & Forbes, 2012).

Studies have shown that the information materials used in the English colorectal cancer program are generally understood by the public (Woodrow et al., 2008). However, lengthy documents with complex and unfamiliar terminology can challenge groups with low levels of literacy and may lead to informational avoidance (von Wagner et al., 2009b). A recent study investigating the comprehensibility of the standard information supplied in the English colorectal cancer screening program (‘Bowel Cancer Screening: The Facts’) found that the numerical risk information resulted in miscomprehension, information processing errors, as well as negative emotional reactions (Smith et al., 2013b).

This study also showed that people tended to understand the information in categorical terms (e.g. reduces risk of dying) rather than interpreting it verbatim (e.g. 16 % relative risk reduction through screening). This observation fits with the medical decision-making theory known as Fuzzy Trace Theory (Reyna, 2008). Fuzzy Trace Theory is a dual-processing theory which suggests that information is encoded in two parallel representations: gist and verbatim. Gist representations are vague, qualitative concepts that capture the ‘bottom-line’ meaning of information. Verbatim representations are precise and quantitative, and capture the literal form. Reyna and colleagues have argued that people have a ‘fuzzy processing preference’; they prefer to process information in gist form and decision-making is improved when doing so (Reyna & Brainerd, 1991, 1995; Reyna & Lloyd, 2006; Reyna, 2011).

Fuzzy Trace Theory also hypothesises that the process of extracting ‘the gist’ from complex information is influenced by literacy and numeracy (Reyna, 2008). This has been demonstrated in a number of studies showing basic literacy skills to be associated with poor comprehension of health information (Berkman et al., 2011; Smith et al., 2012b; Wilson et al., 2010; Wolf et al., 2012). It has been therefore suggested that pre-formulated gist-based information may improve gist extraction, reduce cognitive burden, and improve public understanding of screening (Elwyn et al., 2011). Two recent randomized controlled trials have demonstrated improved comprehension and sustained health outcomes when using Fuzzy Trace Theory intervention strategies that emphasize appropriate gist representations (Reyna & Mills, 2014; Wolfe et al., 2014). Fuzzy Trace Theory therefore provides an elegant theoretical model on which to base a cancer communication intervention that aims to reduce socio-economic inequalities in screening.

We have previously reported on the development of a ‘Gist-based’ colorectal cancer screening information leaflet (Smith et al., 2013c). The information was designed using techniques in keeping with the Fuzzy Trace Theory model. For example, numerical information was presented categorically or using verbal descriptions to provide an evaluative label (i.e. gist) of the number (e.g. most people [98 out of 100]). Gist-based processing was further encouraged by removing information deemed ambiguous or non-essential in our previous studies (Smith et al., 2013b, c). ‘The Gist’ leaflet was evaluated in a small sample of individuals purposively recruited from geographic areas where literacy levels are low. They found ‘The Gist’ leaflet easy to understand, and it had a higher Flesch reading ease score than the standard colorectal cancer screening information ‘The Facts’ booklet (‘The Gist’ = 84.5, ‘The Facts’ = 62.4). To ensure that the process of informed decision-making would still be met for invitees to colorectal cancer screening (Austoker et al., 2012; Ramirez & Forbes, 2012), the gist leaflet was designed to supplement, rather than replace the existing booklet. This raises the possibility that the public will be overloaded with information, which contravenes principles of Fuzzy Trace Theory and the idea that ‘less is more’ when presenting health information (Peters et al., 2013). This may be a particular problem for low numeracy groups (Peters et al., 2007). However, including the existing booklet was necessary to accommodate health system requirements and represents a compromise for using psychological theory within the constraints of an organized screening program.

This study used a randomized controlled trial design to compare socio-cognitive outcomes with ‘The Gist’ leaflet as a supplement to standard information (Gist + Facts) and standard information alone (Facts). Interactions with levels of numeracy were also examined. We hypothesized that ‘The Gist’ leaflet would increase knowledge and screening intentions; and that the difference between conditions would be stronger among low numeracy individuals.

## Methods

### Study design

A multi-center parallel randomized trial design was used. Participants were allocated 1:1 to two groups (‘Facts only’, ‘Gist + Facts’). The study was registered as a trial on the ISRCTN database (ISRCTN62215021) and given ethical approval in February, 2012.

### Participants and setting

General Practices in the North of England were identified. Using the Index of Multiple Deprivation (IMD; a neighbourhood deprivation score based on several socioeconomic markers), three deprived practices and one affluent practice were recruited. IMD is a well-validated marker of socioeconomic status and is linked to colorectal cancer screening uptake (Robb et al., 2010; von Wagner et al., 2011, 2009a). Scores range from 0 (most affluent) to 88 (most deprived). The IMD scores for the practices used in this study were: Liverpool A (77.3), Liverpool B (37.6), Manchester (43.6) and Stockport (10.8).

Staff at the practices produced a list of all men and women aged between 45 and 59.5 years. This age group would not yet have been invited to colorectal cancer screening and therefore had no direct experience with the procedure or the information materials. GPs were invited to exclude patients who had severe cognitive impairments, were vulnerable (e.g. recent diagnosis of significant illness), were under colorectal cancer surveillance, or who were registered as not speaking English.

### Randomisation and blinding

Eligible patients were randomized to intervention or control groups, with all members of a household allocated to the same study group to limit contamination. Software was used to generate a restricted randomization sequence for participant group allocation. Blocking was used to ensure evenly balanced group sizes, which limits the unpredictability of randomization, but this bias was reduced by the use of random blocks (Moher et al., 2010). A researcher (SS) performed the mail-out of study materials from the practice. Group allocation was not concealed at any stage after the random sequence was generated. It was not possible to be blind to the group allocation at data entry or analysis stages because the question related to the acceptability of ‘The Gist’ leaflet was only included for ‘The Gist’ study group. The color of the questionnaires given to the two study groups was also different. Participants were not aware of a comparator group. Randomization occurred prior to consent, which was assumed based on the return of a completed questionnaire.

### Study groups

#### ‘The Facts’ only group

Each participant was provided with a study invitation letter from their GP, a questionnaire, and an example ‘screening pack’ consisting of an NHS-marked envelope with a mock NHS screening invitation letter (watermarked ‘example’) and the standard patient information booklet (‘Bowel Cancer Screening: The Facts’). ‘The Facts’ booklet is 16 pages long and has a Flesch reading score of 62.4 (equivalent of a 13–15 year reading age). The packs were as similar as possible to a real screening invitation to increase ecological validity. Reminders were sent to non-responders after approximately 3 weeks.

#### Gist + Facts group

This group was sent the pack as described above and in addition, ‘The Gist’ leaflet (see online appendix or Smith et al., 2013c for the design process). ‘The Gist’ leaflet is three pages long, and it has a Flesch reading score of 84.5 (equivalent of a 9–11 year reading age). The leaflet was designed to reduce the cognitive burden when making a screening decision by informing the public about colorectal cancer and highlighting that screening is an efficacious way of reducing their risk from the disease. In keeping with informed decision-making standards in the UK, the leaflet did not convey persuasive messages (Ramirez & Forbes, 2012).

Best practice guidelines from the fields of cognitive psychology, information design, and health literacy were used to complement the principles of Fuzzy-Trace Theory during the design process. Numerical information was reduced where possible, but the integrity of the bottom-line meaning of the information was maintained. Consideration of what was the most appropriate ‘gist’ to be conveyed by ‘The Gist’ leaflet was made by experts in the field of colorectal cancer screening. This included the study authors, Specialist Screening Practitioners, directors of the National Bowel Cancer Screening Programme, and an epidemiologist specialising in colorectal cancer screening. In keeping with the ‘less is more’ approach, concepts (e.g. the adenocarcinoma sequence) were removed and only essential information needed to make a screening decision was included (Peters et al., 2013). Messages guided the reader through the information booklet and ‘sign-posted’ where more information could be found. Respondents in both study groups were encouraged to read all of the information in their study pack.

### Measures

#### Gist knowledge

Knowledge was assessed using a method which captures whether individuals have understood the ‘gist’ of the information (Smith et al., 2012a; Tait et al., 2010a, b). Nine items reflecting ‘core’ knowledge outlined by the General Medical Council’s screening guidelines (General Medical Council 2008) and reviews on screening knowledge measures (Mullen et al., 2006; Smith et al., 2012a), were developed. The information to answer these questions was available in both information booklets. ‘True or false’ options, along with ‘do not know’, were provided for each item. ‘Don’t know’ responses were classified as incorrect. One point was given for a correct response, and a total score was calculated. A threshold of 5 (55.5 %) was used to define ‘adequate’ gist knowledge (Smith et al., 2012a). The scale was reliable (α = .73).

#### Intention to be screened

Screening intention was assessed with the item: ‘Imagine you have just turned 60 and have received the bowel screening test kit (FOB test kit) in the post, would you do the test’ (Atkin et al., 2010). Responses options were ‘yes, definitely’, yes, probably, probably not, ‘definitely not’. For these analyses, the ‘yes, definitely’ response was used as a marker of high intention.

#### Participant demographic characteristics

GP records were used to identify the age, gender, number of individuals in a household, and deprivation score of the patient’s home address. These records were used when comparing responders and non-responders. The questionnaire included items on age, gender, marital status, ethnicity, employment status, and education.

#### Numeracy

Numeracy was assessed using the item ‘Which of the following numbers represents the biggest risk of getting a disease?’, ‘1 in 100’, ‘1 in 1,000’, or ‘1 in 10’ (Lipkus et al., 2001). Participants are scored as either correct (higher numeracy) or incorrect (lower numeracy). This item is included within the nationally representative US study, the Health Information and National Trends Survey (HINTS). In the HINTS study, over 20 % of the population were classified as having lower numeracy (Nelson et al., 2013).

#### Acceptability of the materials

Participants were asked to indicate whether they had read the leaflets they were sent, with options of: ‘No’, ‘I have read part of it’, ‘I have read it all’, ‘I have read it all more than once’ (Olamijulo & Duncan, 1997). For analysis we grouped together those who reported reading all of the information at least once.

### Sample size

This study aimed to detect a 5 % difference in the proportion of participants reporting a high level of intention between the study groups. To detect this size of effect (w = .12), 818 respondents were needed assuming 80 % power and *p* = .05.

### Analysis

Respondents were compared with non-respondents using GP data on gender, age, deprivation and number of people in the household using Chi square and t-tests as appropriate.

Analysis included all individuals returning a questionnaire with primary or secondary outcome data. The extent to which participants read the assigned information materials was monitored using descriptive statistics and Chi square. Study outcome variables were described using means (M), standard deviations (SD) and percentages where appropriate. Differences between intention and gist knowledge between the study groups were assessed using the Chi square test, and Analysis of Variance (ANOVA). To investigate condition by numeracy interactions, logistic regression and ANOVA was used. Data were analysed using SPSS version 21.

### Missing data

Missing intention data (.2 %; n = 2) were considered to be missing at random justifying the use of pairwise deletion. The remaining missing data were considered to be missing not at random. Missing gist knowledge data were considered to be missing not at random because most participants attempted 5 or more items out of 9 (99.4 %; n = 958). The remaining individuals (.6 %; n = 6) did not answer any knowledge items and were therefore excluded for all gist knowledge outcomes. Individuals with a portion of missing knowledge data (3.2 %; n = 31) were dealt with by transforming total scores to account for the number of items that participants responded to. For example, if a participant answered 8 out of 9 questions, their total would be computed, divided by 8 and then multiplied by 9 to provide a score from 0 to 9. Missing data for the acceptability of the booklets were minimal (n = 12; 1.2 %), and considered to be missing not at random because these individuals had mostly completed the intention (92 %; n = 11) or knowledge (100 %; n = 12) items, and none had provided an open-text comment about either booklet. Absence of a response on this item therefore suggested they had not read their allocated information materials, and they were coded as such. Sensitivity analyses excluding these individuals were done and yielded similar results. More of the numeracy data were missing (n = 101; 10.5 %). Numeracy data were considered to be missing not at random, as most of these respondents had data for knowledge (94 %; n = 95) and intention (100 %; n = 101), suggesting they had chosen to skip the numeracy item. Knowledge scores for participants with missing data were comparable to those with low numeracy, justifying why we coded them as low numeracy. Sensitivity analyses were done excluding individuals with missing data and yielded similar results.

## Results

**Fig. 1:**
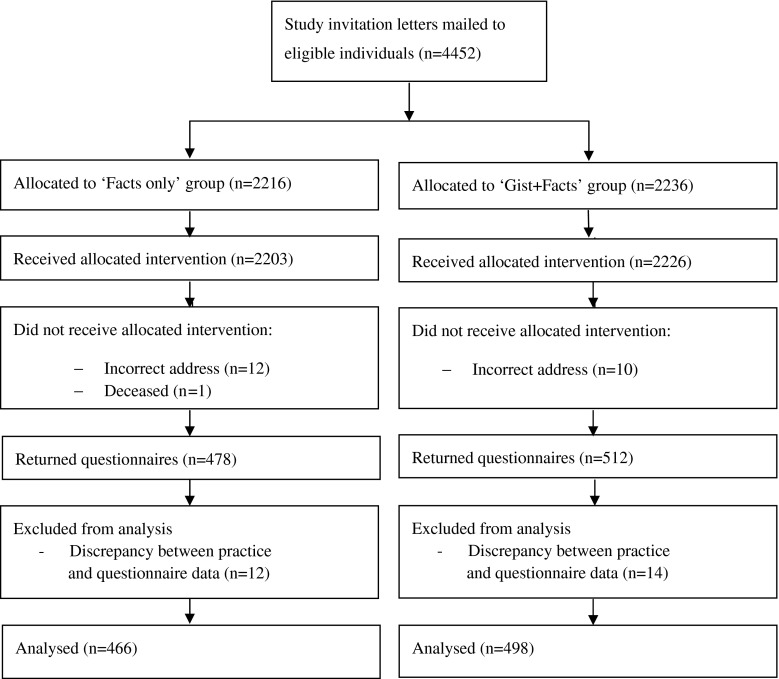
Consort diagram

The study ran between July, 2012 and March, 2013, with questionnaire return up to May, 2013. Individuals (n = 4,452) were randomized by household (n = 3,706), with 2,216 allocated to ‘The Facts’ group and 2,236 to ‘Gist + Facts’ group (see Fig. 1). A total of 3,631 (81.6 %) individuals were sent a reminder (‘Facts only’ group = 1,808 [81.6 %]; ‘Gist + Facts’ group = 1,823 [81.5 %]) approximately three weeks after the initial invitation [median = 22 days (range = 22–41 days)]. Twenty-three invitations were returned not delivered.

Questionnaires were returned by 1,269 individuals, of which 964 were at least partially completed, providing a cooperation rate of 21.9 % following American Association for Public Opinion Research guidelines (The American Association for Public Opinion Research 2011). Questionnaire data on age and gender were compared with practice records and people were excluded if there were discrepancies (n = 26). The cooperation rate varied between the practices, with the most affluent practice (Stockport) having a noticeably higher response [Liverpool a (18.1 %), Manchester (13.0 %), Liverpool b (19.6 %), Stockport (31.8 %)]. These differences were statistically significant (χ^2^(3) = 128.76, *p* < .001).

GP records indicated that the characteristics of the study groups were comparable (see Table 1). Responders were significantly more likely than non-respondents to be female (χ^2^(1) = 16.09, *p* < .001), older (t(4,401) = 6.16, *p* < .001), from an affluent neighbourhood (χ^2^(1) = 115.07, *p* < .001), and be in a home with two or more invitees (χ^2^(1) = 4.05, *p* = .044).

**Table 1:** Characteristics of randomized individuals using General Practice records (n = 4,452)

	All (%) n = 4,452	‘Facts only’ group (%) n = 2,216	‘Gist + Facts’ group (%) n = 2,236
*Gender*			
Male	2,420 (54.5)	1,194 (53.9)	1,226 (54.8)
Female	2,032 (45.6)	1,022 (46.1)	1,010 (45.2)
*Number in household*			
1	2,984 (67)	1,476 (66.6)	1,508 (67.4)
2	1,400 (31.4)	714 (32.2)	686 (30.7)
3	60 (1.3)	22 (1.0)	38 (1.7)
4	8 (.2)	4 (.2)	4 (.2)
*IMD score quintiles*			
1 (low deprivation)	996 (22.4)	473 (21.4)	523 (23.5)
2	794 (17.9)	412 (18.7)	382 (17.1)
3	930 (21.0)	462 (20.9)	468 (21.0)
4	834 (18.8)	420 (19.0)	414 (18.6)
5 (high deprivation)	884 (19.9)	441 (20.0)	443 (19.9)
Age+	51.1 (4.1)	51.2 (4.1)	51 (4.2)

+ Mean and standard deviation reported

Questionnaire data indicated that a high proportion of participants were married (66.9 %), white (83.8 %), in employment (72.2 %) and had either some formal education (49.9 %) or a degree level education (36.5 %) (see Table 2). The sample was well-distributed by gender (51.4 % female) and age group (45–49, 32.7 %; 50–54, 34 %; 55–59, 33.3 %). A high proportion answered the numeracy item incorrectly (35.3 %) or did not provide an answer (10.5 %).

**Table 2:** Participant characteristics for respondents using questionnaire data

	N (valid %)
*Gender*	
Male	466 (48.6)
Female	493 (51.4)
*Age*	
45–49	313 (32.7)
50–54	325 (34)
55–59	319 (33.3)
*Marital status*	
Married	640 (66.9)
Unmarried	317 (33.1)
*Ethnicity*	
White	799 (83.8)
Black	42 (4.4)
South Asian	58 (6.1)
Other	55 (5.8)
*Education*	
No formal education	128 (13.6)
Some formal education	471 (49.9)
Undergraduate or higher	345 (36.5)
*Employment status*	
Employed	689 (72.2)
Unemployed	95 (10.0)
Full-time homemaker	44 (4.6)
Retired	37 (3.9)
Student	5 (.5)
Disabled	84 (8.8)
*Numeracy*	
Correct	523 (54.3)
Incorrect	340 (35.3)
Missing	101 (10.5)

N may not round to 964 due to missing data

Respondents had high knowledge (M = 7.70, SD = 1.74 out of a possible 9) and a large proportion (93.1 %) were classified as having ‘adequate’ gist knowledge. Knowledge was high for most items (Table 3). However, respondents were less likely to correctly answer the items, ‘Bowel cancer is a common cancer in people over 60’ (78.0 % correct) and ‘The FOB test can miss bowel cancer’ (68.5 % correct). The ‘Gist + Facts’ group had a marginally higher mean score than the ‘Facts only’ group (M = 7.81, SD = 1.64 vs. 7.59, SD = 1.83, respectively; t(924.7) = −1.90, *p* = .057). Individuals in the ‘Gist + Facts’ group were more likely to have adequate knowledge (95.2 %) than the ‘Facts only’ group (90.9 %; χ^2^(1) = 6.74, *p* = .009) (see Table 4). There were larger differences between the study groups for the items, ‘People aged 60–74 years are sent the FOB test’ (7.7 % difference), ‘Doing the FOB test lowers the risk of dying from bowel cancer’ (3.6 % difference), and ‘People only need to do the FOB test once in their life’ (3.6 % difference) (Table 3). Low numeracy individuals had poorer knowledge than the high numeracy group overall (M = 7.28, SD = 1.96; M = 8.05; SD = 1.44, respectively; t(783.2) = 6.77, *p* < .001) and were less likely to have adequate knowledge [89.0 vs. 96.6 % (7.6 % diff); χ^2^(1) = 21.34, *p* < .001]. There was no significant group by numeracy level interaction for either the continuous measure (F(1, 954) = .68, *p* = .625) or having adequate knowledge (OR .42, 95 % CI .13–1.30, *p* = .130). This suggests that the knowledge improvements observed were experienced equally across numeracy groups, and the low numeracy group did not disproportionately improve.

**Table 3:** Descriptive differences between study groups for each knowledge item

	% Correct			
	All	‘Gist + Facts’ group	‘Facts only’ group	Difference (%)
Doing the FOB test lowers the risk of dying from bowel cancer (true)	87.6	89.3	85.7	3.6
The FOB test is done at home (true)	94.5	95.2	93.7	1.5
Most people who do the FOB test will receive an abnormal result (false)	82.4	82.3	82.5	−.2
Only women are sent a FOB test (false)	95.0	95.8	94.2	1.6
Bowel cancer is a common cancer in people over 60 (true)	78.0	78.8	77.1	1.7
People only need to do the FOB test once in their life (false)	89.6	91.3	87.7	3.6
The FOB test can miss bowel cancer (true)	68.5	68.5	68.4	.1
People with an abnormal result always have cancer (false)	88.8	89.7	87.9	1.8
People aged 60–74 years are sent the FOB test (true)	83.0	86.7	79.0	7.7

**Table 4:** Differences between study groups on outcome measures

Variable	‘Gist + Facts’ group	‘Facts only’ group	Significance
	%	%	
Intention	75.7	73.8	χ^2^(1) = .45, *p* = .50
Gist knowledge	95.2	90.9	χ^2^(1) = 6.74, *p* = .009

A large proportion of the sample said they would *‘definitely’* (74.7 %) or *‘probably’* (22.9 %) participate in screening, and very few reported that they would *‘probably not’* (1.6 %) or *‘definitely not’* (.8 %) participate. Due to the small number of individuals indicating that they would not participate in screening (Gist n = 13; Facts n = 10), we collapsed the bottom three categories and compared these responses to ‘definite’ intenders. There were no significant differences between the two study groups in the proportion of individuals who definitely intended to participate (χ^2^(1) = .45, *p* = .50) (see Table 4). Low numeracy individuals were less likely to say they would *‘definitely’* participate in colorectal cancer screening [71.2 vs. 77.7 % (6.5 %); χ^2^(1) = 5.40, *p* = .020]. There was no significant group by numeracy level interaction for the intention outcome (OR 1.02, 95 % CI .57–1.84, *p* = .936). This suggests that the effect of ‘The Gist’ leaflet on intention was equal across numeracy groups.

In the whole sample, 81.7 % reported reading all of the information at least once, but those with poor numeracy were less likely to report this [74.4 vs. 88.0 % (13.6 % diff); χ^2^(1) = 29.56, *p* < .001]. There was no significant group by numeracy level interaction in terms of self-reported reading of the information (OR 1.37, 95 % CI .69–2.72, *p* = .367).

**Fig. 2:**
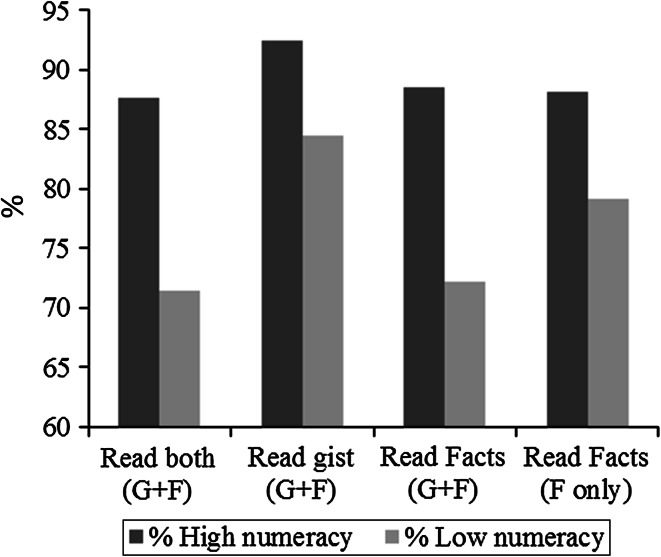
Proportion of participants who reported reading at least some of their allocated materials by numeracy group

Overall, the ‘Gist + Facts’ group were marginally less likely to report reading the materials than the ‘Facts only’ group (79.7 vs. 83.9 %; χ^2^(1) = 2.83, *p* = .093). Within the ‘Gist + Facts’ group, comparisons between the two booklets suggested participants were more likely to report reading ‘The Gist’ leaflet (88.6 %) than ‘The Facts’ booklet (80.5 %). Within the ‘Gist + Facts’ group, compared with the high numeracy group, participants with low numeracy were slightly less likely to report reading ‘The Gist’ leaflet [84.5 vs. 92.5 % (8.0 % diff); χ^2^(1) = 7.86, *p* = .005], and even less likely to report reading ‘The Facts’ booklet (72.2 vs. 88.5 % (16.3 % diff); χ^2^(1) = 21.07, *p* < .001] (Fig. 2). There was also a significant difference in reported reading between the low and high numeracy groups in ‘The Facts’ only group [79.1 vs. 88.1 % (9.0 % diff); χ^2^ = 8.56, *p* = .003] (see Fig. 2).

## Discussion

This study reports the effects on knowledge and screening intentions of adding a supplementary Gist-based leaflet to the standard information materials used in the English NHS colorectal cancer screening program in a deprived community sample. Provision of ‘The Gist’ leaflet to the existing materials led to increased knowledge but had no effect on screening intention.

We designed a theory-based intervention that could be delivered cheaply and efficiently, without the need for healthcare professional input. Preliminary testing of ‘The Gist’ leaflet showed that it was comprehensible and acceptable to a low literacy audience (Smith et al., 2013c). To adhere to agreed standards of informed decision making in the UK screening program, ‘The Gist’ leaflet had to be added to the existing information as opposed to being used in a ‘standalone’ format (Ramirez & Forbes, 2012). We have previously shown that ‘The Facts’ booklet can be difficult to process, particularly with regard to the medical terminology and numerical risk information (Smith et al., 2013b). It is therefore possible that our approach of providing alternative low literacy information may have been diluted by the presence of ‘The Facts’ booklet.

A small US trial in Federally Qualified Health Centers serving underserved populations has recently reported substantial increases in colorectal cancer screening uptake by using low literacy educational materials and healthcare professional support (Davis et al., 2013). Several others have also reported the effectiveness of meeting the literacy demands of lower socioeconomic status groups in a cancer screening setting (Christy et al., 2013; Ferreira et al., 2005; Miller et al., 2011). These studies suggest that health communication tools are an effective way of reducing colorectal cancer screening disparities. However, these trials were resource-intensive (i.e. they involved healthcare professional support) and the communication materials were designed to replace rather than supplement existing information. This emphasises the importance of evaluating our Gist leaflet as standalone information.

In this study, we tested the assumption of Fuzzy Trace Theory that the process of gist extraction from health information will be easier for low numeracy groups with gist-based information because of the reduced cognitive burden (Reyna, 2008). In support of Fuzzy Trace Theory, data indicated that ‘Gist + Facts’ group had higher levels of adequate knowledge than ‘The Facts’ group. As shown by the grey bars in Fig. 2, low numeracy individuals were most likely to report reading ‘The Gist’ leaflet, and levels were comparable to those with high numeracy. It was interesting to note that more people achieved adequate knowledge than reported reading their allocated materials, suggesting that some respondents already possessed an adequate level of knowledge about colorectal cancer screening. One explanation is that the knowledge items were too simple, and the effectiveness of the ‘The Gist’ leaflet should be tested using more challenging items. Alternatively, the threshold suggested by Smith et al. may be too lenient for testing cancer screening information materials (Smith et al. 2012a). Nonetheless, our findings provide some support for Fuzzy Trace Theory by showing that gist-based information is preferred and that it increases the ease through which information is processed (Reyna, 2008). It also provides evidence that ‘The Gist’ leaflet may be a simple but effective way of increasing public engagement with cancer communication materials.

There were similar improvements to gist knowledge across high and low numeracy groups. This is an important finding as health communication interventions can inadvertently exacerbate communication inequalities (Boxell et al., 2012; Viswanath, 2005). ‘The Gist’ leaflet could therefore be incorporated into the screening program without fear that it would increase inequalities. However, it was disappointing that our moderation analyses indicated that differences between low and high numeracy groups were not reduced by the inclusion of ‘The Gist’ leaflet. It is possible that our interpretation of how to present information according to Fuzzy Trace Theory was too subtle; meaning the ability to extract the gist of information was not simple enough. This situation is likely to have been made worse by the fact that they received ‘The Facts’ booklet too. However, most UK adults want *full* information about the risks and benefits of colorectal cancer screening (Waller et al., 2012), and background knowledge is often needed to extract a meaningful gist (Reyna, 2012). Considering that such knowledge is often lacking in medical scenarios, simplifying the information materials further is likely to be challenging without compromising informed decision-making (Austoker et al., 2012; McCaffery et al., 2013; Ramirez & Forbes, 2012).

The finding that participants with low numeracy were less likely to read their allocated materials supports evidence that lower socioeconomic status groups tend to avoid information about cancer (McCloud et al., 2013; von Wagner et al., 2009b). This has implications for health care providers and organisations who communicate with the public about cancer prevention and control. It is often assumed that if information is made easier to interpret, it will motivate the public to engage with it; but complementary interventions may be needed to engage the public and use of ideas from the fields of health literacy and patient activation could provide novel strategies to address communication inequalities within colorectal cancer screening programs (Smith et al., 2013a).

This study has limitations. The most serious was the low response rate (22 %); which was lower than our similar studies recruiting from primary care (Robb et al., 2008; Wardle et al., 2003); but these had not focused on deprived areas. Response was higher in the most affluent practice, and at an individual level responders were more likely to be female, older, from an affluent neighborhood and be living in a household with two or more invitees. The study population should therefore be considered a less deprived sub-sample of those that were invited. Equally serious was the underrepresentation of respondents who did not wish to be screened; with over 95 % of our respondents expressing positive attitudes and an inclination towards colorectal cancer screening. This reduced the chance of detecting any impact of ‘The Gist’ information. Similar Figs. (85–95 %) have been reported in previous community-based studies (Robb et al., 2008; Wardle et al., 2003).

The focus on individuals who had not previously been invited for colorectal cancer screening was both a strength and a limitation. On the positive side, participants were not biased by past behavior (Murphy et al., 2013). However, one possibility for the null effects on intention is that colorectal cancer screening was not sufficiently salient because of the age of the participants and the hypothetical scenario (Myers et al., 1998; Tiro et al., 2005; Vernon et al., 2001). Respondents’ age may also partly explain the high intentions to be screened at age 60. Construal-Level Theory suggests that people considering the possibility of being screened in the distant future are less likely to construe the behavior in concrete terms, with all its practical disadvantages (Trope & Liberman, 2010; von Wagner et al., 2010). Recent research suggests that the process through which psychological distance effects decision-making is mediated through changes to gist representations, consistent with Fuzzy Trace Theory (Fukukura et al., 2013).

The study made use of a proxy marker of colorectal cancer screening behavior, i.e. screening intention, and although it is a valuable indicator (Cooke & French, 2008), it may not be nuanced enough to examine the subtle effects of different types of information; as was apparent from the high level of intention. The other measurement limitation was the use of a single numeracy item taken from the Health Information and National Trends Survey, which will have lacked the discriminant validity of a full numeracy scale (Lipkus et al., 2001).

This multi-center parallel randomized controlled trial found that a supplementary gist-based leaflet increased knowledge but did not increase intention to participate in colorectal cancer screening; but this finding has to be tempered by the very high intention levels among the study respondents. Future studies should examine gist information presented alone rather than alongside highly detailed information, and examine the cost-effectiveness of testing these strategies alongside healthcare professional support.

## Electronic supplementary material

Below is the link to the electronic supplementary material.

Supplementary material 1 (PDF 325 kb)
