# Supplementary material for: The effect of a supplementary (‘gist-based’) information leaflet on colorectal cancer knowledge and screening intention: a randomized controlled trial
Source: J Behav Med. 2014 Sep 25;38(2):261–72. doi: 10.1007/s10865-014-9596-z (PMC4353886; doi:10.1007/s10865-014-9596-z)

DEVELOPED BY UNIVERSITY COLLEGE LONDON
WITH FUNDING FROM
THE NATIONAL INSTITUTE FOR HEALTH RESEARCH

NHS
National Institute for
Health Research

NHS Bowel Cancer Screening Programme: The Essentials

- Bowel cancer is the third most common cancer
- The FOB test spots hidden signs of bowel cancer early
- Doing the FOB test every 2 years lowers the risk of dying from bowel cancer
- Men and women aged 60-74 are sent the FOB test to do at home

Would you like to know more? See inside for details

How does the FOB test work?

- The FOB (Faecal Occult Blood) test checks for tiny amounts of blood in stools (poo) that cannot be seen by the eye
- Blood in stools can be a sign of bowel cancer
- An FOB test kit is sent to your home
- The FOB test is easy to do
- You do the FOB test at home by putting small amounts of stool onto a test kit
- You send the test kit back to the laboratory in a special freepost envelope

What happens after you've done the FOB test?

- You get your FOB result through the post within 2 weeks
- Most people (98 out of 100) get a normal result
- If you have a normal result you will be sent another FOB test every 2 years up to age 74
- A small number of people (2 out of 100) get an abnormal result
- If you get an abnormal result, you will get an appointment to talk about further testing
- For most people, the follow-up test will show there is no bowel cancer
- If bowel cancer is found, it is likely to be at an early stage where treatment is more successful

How accurate is the FOB test?

- Doing the FOB test every 2 years lowers the risk of dying from bowel cancer
- Like all screening tests, the FOB test is not 100% accurate
- Bowel cancer can be missed if there is no bleeding at the time the FOB test is done

Where can I get more information?

For more information see the enclosed leaflet:

'Bowel Cancer Screening: The Facts'

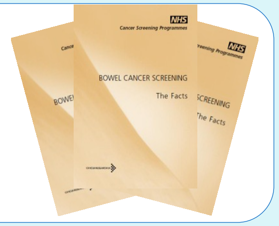

Supplement: Supplementary file 1 — Supplementary material 1 (PDF 325 kb) [file 10865_2014_9596_MOESM1_ESM.pdf]
